# Effects of Erythropoietin on Electrocardiogram Changes in Carbon Monoxide Poisoning: an Experimental Study in Rats 

**Published:** 2012

**Authors:** Mitra Asgharian Rezaee, Seyed Adel Moallem, Mohsen Imenshahidi, Mahdi Farzadnia, Amir Hooshang Mohammadpour

**Affiliations:** a *Department of Pharmacodynamics and Toxicology, School of Pharmacy, Mashhad University of Medical Sciences, Mashhad, Iran. *; b *Medical Toxicology Research Center, Mashhad University of Medical Sciences, Mashhad, Iran. *; c *Department of Pathology, School of Medicine, Imam Reza Hospital, Mashhad University of Medical Sciences, Mashhad, Iran. *; d *Pharmaceutical Research Center, Mashhad University of Medical Sciences, Mashhad, Iran. *

**Keywords:** Carbon monoxide poisoning, Cardiotoxicity, Electrocardiogram, Erythropoietin

## Abstract

The aim of this study was to define the electrocardiogram (ECG) changes following the moderate to severe CO intoxication in rats, and also evaluating the effect of erythropoietin (EPO) on observed cardiac disturbances. The growing literature on erythropoietin effect on cardiac ischemia led us to question its effect on cardiotoxicity due to the carbon monoxide poisoning.

Wistar rats were exposed to three different concentrations of CO (250 PPM, 1000 PPM or 3000 PPM). EPO was administrated (5000 IU/Kg, intraperitoneal injection) at the end of CO exposure and then the animals were re-oxygenated with ambient air. Subsequently ECG recording, heart rate and carboxyhemoglobin values were evaluated.

ECG changes following the CO intoxication included ST segment elevation and depression, T wave inversion and first-degree AV block. Ischemic ECG changes reduced significantly in EPO-treated animals.

In the present study, for the first time, EPO was investigated for the management of cardiac complications due to the CO poisoning. Our results showed that EPO could inhibit ischemic changes of ECG after the CO poisoning.

## Introduction

Carbon monoxide (CO) intoxication is one of the most common and complicated cases in poisoning centers. It is associated with more than half of fatal intoxication worldwide (1, 2). Cardiac disorders have been demonstrated as one of the major CO poisoning consequences (3). The most common cardiovascular disturbances in acute CO poisoning are tachycardia, dysrhythmia, hypotension, ischemia, infarction and in more severe cases, cardiac arrest (2, 4). The major mechanisms of CO toxicity include CO binding to heme proteins, the change in dissociation of oxyhemoglobin and subsequently tissue hypoxia. Tissue hypoxia and also direct toxicity of CO can lead to stress responses and cell injury especially in cardiac and neurologic tissues (2, 5, 6). Ischemia and myocardial injury are common in moderate to severe CO intoxication (5). Data from human studies have shown ischemic ECG changes following the CO poisoning including T wave flattening or inversion, ST segment elevation and depression, QT prolongation, atrial fibrillation and intraventricular block (3, 7). The most reported ECG changes were T wave abnormality and ST segment changes (elevation and depression) (3, 4, 7). In addition, ECG ischemic changes including PR prolongation and AV block have been reported in animal models of CO intoxication (7). Ventricular tachycardia and ventricular fibrillation were demonstrated following severe CO poisoning in rat (8).

Recently, new therapeutic effects of EPO have been introduced. EPO, a hematopoietic hormone produced by the kidney in response to hypoxia, has been investigated as anti-ischemic agent (9). This new non-erythropoietic effect of EPO was considered when EPO receptors were found in non-hematopoietic sites such as: endothelial cells, neurons, cardiac myocytes and vascular smooth cells. There are numerous studies showing the protection effect of EPO against ischemia especially in the cardiac and neurologic systems (9, 10).

As previously well-described, ischemic insult results in apoptosis and necrosis cell death. It is believed that the anti-oxidative and anti-apoptotic properties of EPO have roles on its anti-ischemic effect (11, 12). Many *in-vivo *and *in-vitro *studies have been carried out to investigate the EPO protective mechanisms. These studies have described several involved signaling pathways including JAK/STAT, PI3/AKT, MAP 42/44 and PKC (9, 10, 13). The outcomes of activating these pathways in cardiac ischemia are ventricular function preservation and infarct size reduction as well as significant prevention of apoptosis (13).

The importance of cardiac toxicity in the morbidity and mortality in CO poisoning, the evidence of cardioprotective effect of EPO and the known mechanisms of CO poisoning and EPO effects led us to hypothesize that EPO could reduce the cardiac injury of CO poisoning. This study was designed to evaluate the effect of EPO on ischemic ECG changes due to the CO intoxication in rat. The results of the present study were indicated for the first time that EPO could prevent ischemic ECG changes of CO poisoning in a rat experimental model. 

## Experimental

*Animals*

Wistar male rats, weighing 200-250 g, were housed in the Animal Center of Buali Research Institute. They were kept under standard conditions (21-23°C temperature, 12 h/12 h light/dark cycle) with free access to food and water. All animals were treated in accordance with the Guidelines for the Care and Use of Laboratory Animals prepared by the National Academy of Sciences and published by the National Institutes of Health (NIH publication no. 85-23, Revised 1996). The study was approved by the Animal Care Committee of Mashhad University of Medical Sciences.

*Experimental groups and study design*

All animals were anesthetized by ketamine/xylazine (100/10 mg/Kg, IP) before CO poisoning and anesthesia was maintained during the experiments at half of the initial dose (14). A preliminary ECG was recorded before the CO exposure. Then, the animals were placed in a 12 L airtight plexiglass container with entrance and exit taps. CO (0.03%) was flowed to the container at a constant flow of 0.5 L/min for 3000 PPM and 0.3 L/min for 1000 and 250 PPM after the adjustment of CO concentration. The CO concentration was monitored continuously with a CO analyzer (TPI.707 carbon monoxide analyzer, Korea). Three different models of CO intoxication were selected for mild, moderate and acute CO intoxication based on previous animal studies (15-17). The difference of these models was in CO concentrations and exposure times. At the end of CO exposure, recombinant human EPO (rhEPO, Pooyesh Draou Co, Iran) at 5000 IU/Kg was injected intraperitoneally according to the other animal studies (11). At this time, the blood sample was taken from the tail vein and the animals were exposed to ambient air. The time course of the experiments was 3 h. Animal groups were as the following: Group 1 (3000 PPM CO group): Five animals were exposed to 1000 PPM of CO for 20 min, then 3000 PPM for 40 min. Afterward, the animals were re-oxygenated with normal air. Group 2 (3000 PPM CO-EPO group): Five animals were exposed to 1000 PPM of CO for 20 min, then 3000 PPM for 40 min. EPO was injected after CO exposure, and then the animals were re-oxygenated with normal air. Group 3 (1000 PPM CO group): Five rats were exposed to 1000 PPM of CO for 40 min, and then were re-oxygenated with exposure to normal air. Group 4 (1000 PPM CO-EPO group): Five rats were exposed to 1000 PPM of CO for 40 min then received EPO and re-oxygenated with normal air. Group 5 (250 PPM CO group): Five rats were exposed to 250 PPM of CO for 90 min then re-oxygenated with normal air. Group 6 (250 PPM CO-EPO group): Five animals were exposed to 250 PPM of CO for 90 min and then received EPO and re-oxygenated with normal air. Group 7 (EPO control group): Five rats received only EPO as control of EPO. Group 8 (control group): Five rats, no CO inhalation and no EPO treatment.

*ECG and heart rate recording*

ECG in lead І and also heart rate were recorded (PowerLab, ADInstrument, Australia) every 40 min during the experiment time, before and during the CO inhalation and also afterwards when the animals were breathing normal air. Subsequently, the ECG was analyzed for changes in ST segment, T wave, AV block, pathologic Q wave and arrhythmia (premature ventricular contraction (PVC), sinus sick syndrome (SSS), ventricular tachycardia and fibrillation and atrial fibrillation).

*Carboxyhemoglobin level assessment*

Blood samples were taken from the tail vein immediately after the CO inhalation, and carboxyhemoglobin levels were measured with the use of a spectrophotometer (jenway 6305, spectrophotometer, UK) (18).

*Statistical analysis*

Data analyses were performed using SPSS version 11.5. Individual groups were assessed with chi-square or Fisher’s exact test. Statistical significance was determined by using One-way ANOVA test for quantitative data. All statistical tests were 2-sided. Differences were considered significant at p < 0.05.

## Results

ECG and heart rate changes were compared among treated and untreated groups and between CO exposed and control groups. Significant ECG changes were observed in the 3000 PPM and 250 PPM groups. ECG changes in CO-intoxicated and EPO-treated animals are shown in Figure 2-5. In addition, the mean ± SD of ST segment and heart rate changes of each group are presented in Table 1. EPO administration didn’t change significantly heart rate after 1 to 2 h compared to untreated groups (Table 1). Carboxyhemoglobin levels following CO intoxication are shown in Table 2. Carboxyhemoglobin levels indicated acute CO poisoning especially after the intoxication by 3000 PPM of CO.

**Table 1:** ST segment changes and heart rate during CO inhalation, 1 and 2 h after re-oxygenation in all groups (mean ± SD).

**Groups**	**Δ ST** **a** (during CO*)	Δ ST (1 h**)	Δ ST (2 h***)	Heart rate (1 h**)	Heart rate (2 h***)
3000 PPM CO	6 × 10-5 ± 2 × 10-5 (p < 0.01b)	-5×10-5±2×10-5 (p < 0.01b)	-6×10-5 ± 3 × 10-5 (p < 0.01b)	179 ± 41.2	221.8 ± 66.5
3000 PPM C(O+ EPO)	4 × 10-5 ± 3 × 10-5 (p < 0.05b)	-4 × 10-5 ± 3 × 10-5 (p < 0.05b)	-3 × 10-6 ± 2 × 10-5 (NS)	168.8 ± 45.2	181.2 ± 22
1000 PPM CO	0	-1 × 10-5 ± 3 × 10-5 (p < 0.05b)	-6 × 10-6 ± 6 × 10-6 (NS)	194 ± 49.6	202 ± 59.8
1000 PPM CO +EPO	0	-2×10-5±1×10-5 (NS)	0	178 ± 28	202 ± 24
250 PPM CO	1 × 10-5 ± 4 × 10-6 (NS)	-2 × 10-5 ± 5 × 10-6 (NS)	-3×10-5±2×10-5 p < 0.05b	248 ± 67	203 ± 54
250 PPM CO+EPO	0	0	0	250 ± 38.3	218 ± 39.9
EPO	0	-4 × 10-6 ± 6 × 10-6 (NS)	0	265 ± 26	238 ± 33
Control	0	0	1 × 10-5 ± 3 × 10-6 (NS)	208 ± 25	200 ± 31

a: ΔST: mean changes of ST segment compare to control; b: p-value statistically calculated with One-Way ANOVA; *: end of CO exposure; **: 1 h after re-oxygenation; ***: 2 h after re-oxygenation; NS: non-significant.

**Table 2:** Carboxyhemoglobin levels following CO intoxication in animal groups

**Groups**	**Mean ± SD**	**Range (%)**
3000 PPM	70 ± 8	60-76
1000 PPM	31 ± 11	19-46
250 PPM	10 ± 5	13-10

*Animals intoxicated with 3000 PPM of CO*

**Figure 1:**
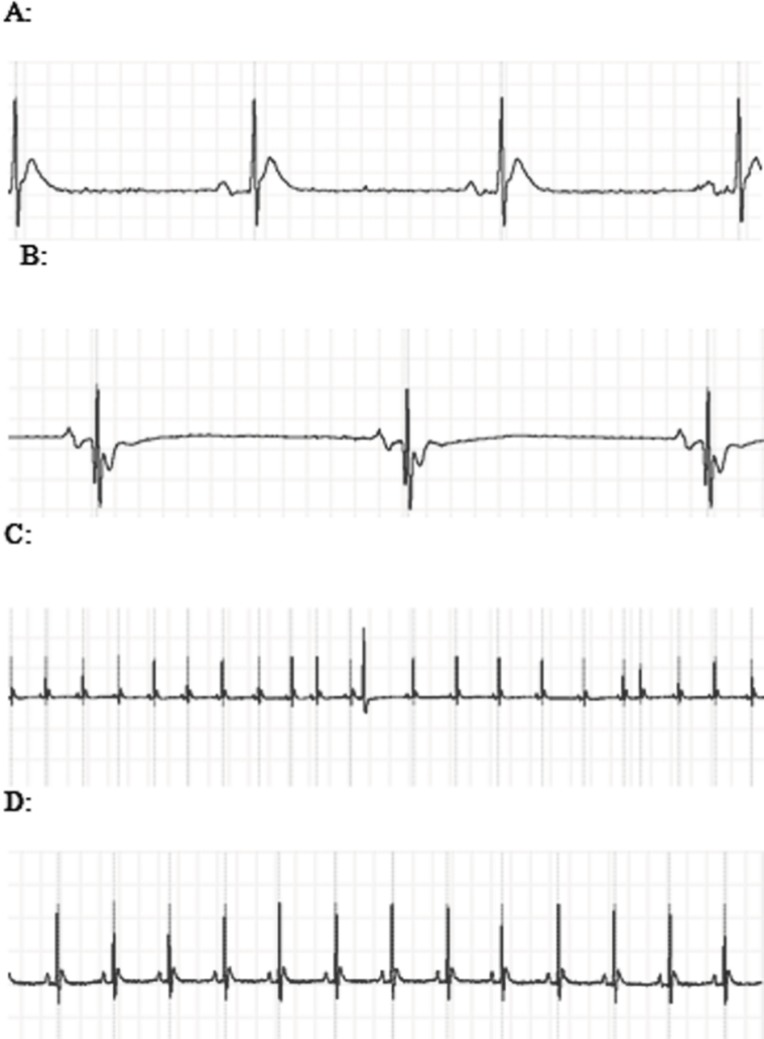
A: The ECG showed ST segment elevation during exposure to 3000 PPM of CO. B: ST segment depression and T wave inversion after 1 h of re-oxygenation following the intoxication with 3000 PPM of CO. C: PVC and sick sinus syndrome (SSS) after intoxication with 3000 PPM of CO. D: Normal

**Figure 2:**
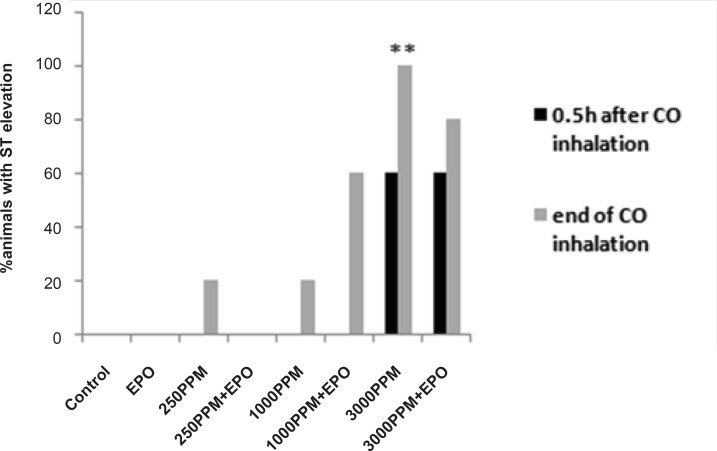
Percent of animals with ST segment elevation during CO exposure, ST elevation presented significantly in animals intoxicated with 3000 PPM of CO (**: p < 0.01 vs. control).

**Figure 3:**
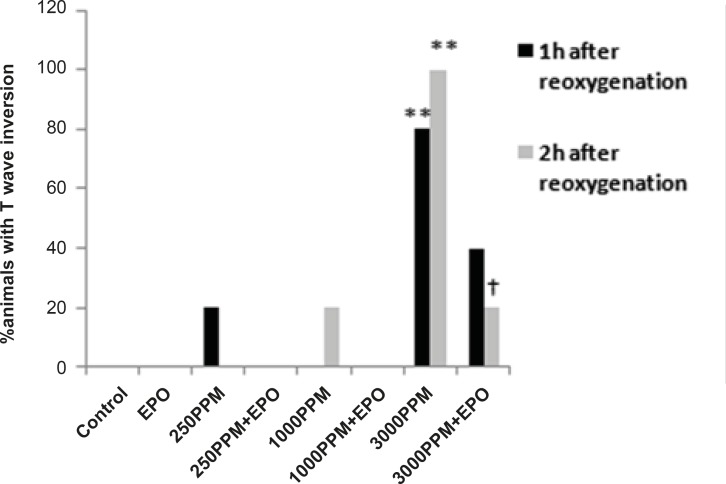
Percent of animals with T wave inversion 1 and 2 h after the re-oxygenation. This change was significant in animals intoxicated with 3000 PPM of CO after 1 h and 2 h of re-oxygenation (**: p < 0.01 vs. control). EPO significantly inhibited T wave inversion 2 h after the re-oxygenation (†: p < 0.05 vs.3000 PPM/2h after the re-oxygenation).

**Figure 4:**
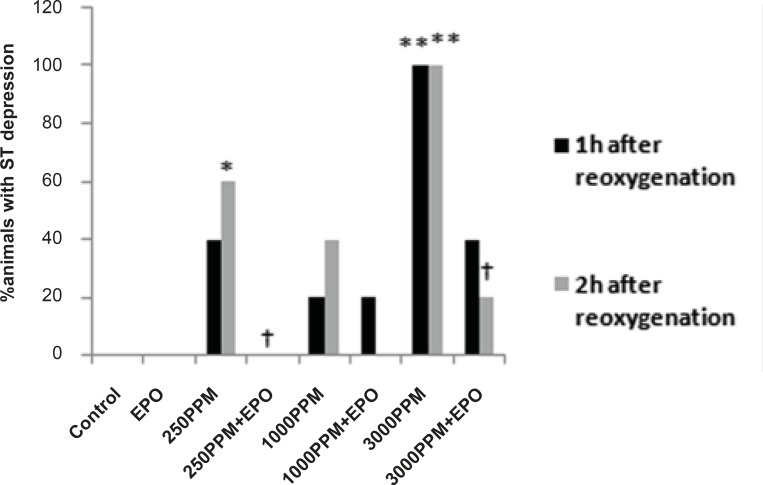
Percent of animals with ST segment depression following 1 and 2 h re-oxygenation. This change was significant in the 3000 PPM group (**: p < 0.01 vs. control) and in the 250 PPM CO group (*: p < 0.05 vs. control). EPO inhibited ST segment depression in both the 3000 (†: p< 0.05 vs. 3000 PPM) and 250 PPM groups (†: p < 0.05 vs. 250 PPM after 2 h re-oxygenation).

**Figure 5:**
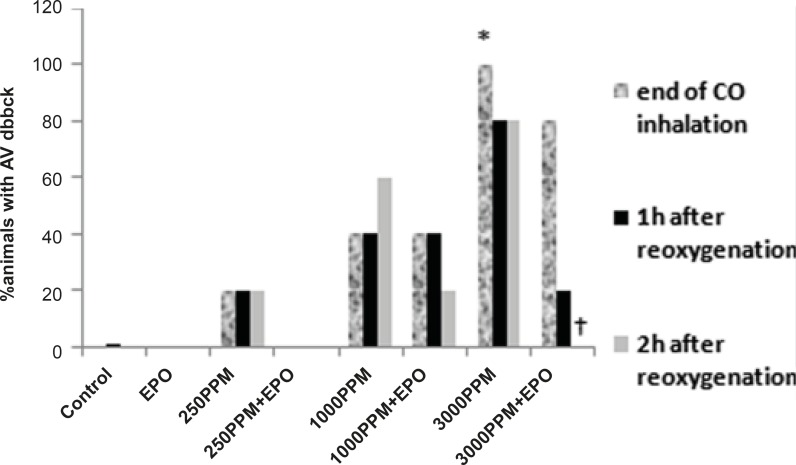
Effects of CO on PR prolongation (percent of animal with prolonged PR). PR prolongation (first degree AV block) was induced significantly following the intoxication with 3000 PPM of CO (*: p < 0.05 vs. control). First degree of AV block reduced significantly in EPO administrated animals, 2 h after the re-oxygenation (†: p < 0.05 vs. 3000 PPM 2 h after re-oxygenation).

ECG changes included ST segment elevation during CO exposure (Figure 1A and Figure 2; p < 0.01), ST depression and T wave inversion, 1 and 2 h after breathing in normal air (Figure 1B and Figures 3 and 4; p < 0.01) and first degree AV block (Figure 5; p < 0.05). In this group, pathologic Q wave was seen in 40% of animals 2 h after the reoxygenation. Besides, arrhythmia of sinus sick syndrome (SSS) was recorded in 40% of animals during the exposure to 3000 PPM. In animals that received EPO, just after CO exposure, T wave inversion was inhibited significantly 2 h after the re-oxygenation in the EPO administrated animals (Figure 3; p < 0.05), and also this inhibition was 50% after 1 h of re-oxygenation. EPO significantly inhibited ST segment depression due to CO exposure compared with the CO group (p < 0.05; Figure 4). ST depression was reduced 60% after 1 h of re-oxygenation in EPO-treated animals. EPO also reduced the first degree AV block significantly 2 h after the re-oxygenation (Figure 5; p < 0.05).*Animals intoxicated with 1000 PPM of CO* ECG changes following CO intoxication were not statistically significant compared to the control group. However, as it can be seen from Figure 3, T wave inversion after 2 h of re-oxygenation was recorded in 20% of animals and EPO treatment completely abolished this inversion. ST segment depression was seen 1 and 2 h after the re-oxygenation in 20% and 40% of animals in this group, respectively (Figure 4). First Av block was recorded at the end of CO exposure, 1 and 2 h after the re-oxygenation in 40%, 40% and 60% of cases, respectively (Figure 5). In EPO-treated animals, ST segment depression, T wave inversion and first degree AV block were completely or partially inhibited in comparison with 1000 PPM CO group (Figures 3, 4 and 5).

*Animals intoxicated with 250 PPM of CO*

ST depression was observed 2 h after the re-oxygenation significantly compared to the control group (p < 0.05; Figure 4) and EPO significantly reduced ST depression (p < 0.05; Figure 4). Other ECG changes ST segment elevation, T wave inversion (after 1 h of re-oxygenation), ST depression (after 1 h of re-oxygenation) and AV block were observed in 20%, 20%, 40% and 20% of animals, respectively (Figures 2-5) and EPO completely inhibited these ECG abnormalities.

Additionally, comparison of ECG changes between three different CO groups indicated that the incidence of ST depression and elevation and first degree AV block were significantly higher in the 3000 PPM group compared with the 1000 PPM group. ST elevation and first degree of AV block were manifested significantly by 3000 PPM of CO in comparison with 250 PPM (p < 0.05). No significant difference was shown in the EPO control group compared to the control. 

## Discussion

In this study, for the first time, we have revealed a major effect of EPO in reducing ischemic ECG changes due to CO intoxication. Our results showed that ECG changes following CO intoxication were dose-dependent and also time-dependent and more ischemic changes were recorded in 3000 PPM CO group comparing with other groups. ST segment elevation, first degree AV block, ST segment depression and T wave inversion were recorded in various CO exposed groups, especially in the 3000 PPM.

The CO concentration and also exposure times in each group were determined in basis of previous animal study for mild, moderate and severe CO intoxication. Our results showed that some ECG changes were statistically significant in the 250 PPM CO group, but not in the 1000 PPM group, whereas, exposure time in 250 PPM CO group was longer than the 1000 PPM CO group (90 min vs. 40 min). This result suggests that in addition to CO concentration, the exposure time is import in CO pathogenesis. ST segment depression, T wave inversion and first degree AV block were significantly inhibited by EPO administration at the end of CO exposure. It is important to emphasize the timing of EPO treatment in this study. Since it is almost impossible to pre-treat CO poisoned patients, we did not intend to evaluate the pretreatment effect of EPO against CO consequences, *i.e. *the timing of EPO treatment was not before or at least during CO exposure, so we administered EPO at the end of CO exposure (the beginning of re-oxygenation).

Other animal studies of CO intoxication have reported that CO exposure at different intensities caused increase in PR interval, QT interval and T wave in a dose-dependent manner (7). Fineschi *et al*.(8) have reported the occurrence of ventricular tachycardia (VT) and ventricular fibrillation (VF) in rats exposed to 2% CO. In their study, the CO concentration (20000 PPM) was much higher than our study. In our examination, the maximum CO concentration was 3000 PPM. It seems that cardiac toxicity and ECG changes in CO poisoning studies are extensively dose and time related which indicate the poisoning severity. This probably explains why ischemic changes were not reported in some similar animal studies (7, 8). It is noteworthy that ST segment depression and T wave inversion in ECG could be considered as the marker of ischemia. Furthermore, impaired conduction in AV could occur as a result of ischemia (19).

CO Cardiotoxicity is associated with hypoxia/ischemia damage. This is explained via hypoxia induced by the carboxyhemoglobin formation and a leftward shift of oxyhemoglobin dissociation curve. More hypoxia is caused by CO binding to other heme proteins such as cytochrome C oxidase and myoglobin (20). Hypoxia results in reactive oxygen species (ROS) formation and oxidative stress and consequently inflammation, cellular necrosis and apoptosis (2). Other factors involved in oxidative stress and cell injury include the increase of heme oxygenase-1 proteins in intracellular levels, activation of hypoxia-inducible factors 1α (HIF-1α) and release of nitric oxide following CO binding to platelet heme proteins (2, 4-6).

Recently, EPO has been investigated extensively as a protective agent against the ischemia and cardiac injury. There are growing evidence indicating that the EPO administration could reduce ischemia and improve cardiovascular function after ischemia/reperfusion (I/R) injury (10-13, 21). Other *in-vivo *studies of cardiac ischemia in rat model indicated that EPO could have acute cardioprotective effect at 5000 IU/Kg with reduction in cardiomyocyte loss, infarct size and apoptosis, and also normalization of hemodynamic function (22-24). In our study, EPO significantly reduced the ischemic changes which are evident in recorded ECG in comparison with the untreated group.

The administration of EPO before ischemia or at the onset of ischemia and/or reperfusion, could reduce or prevent the ischemia/reperfusion injury and improve the cardiac function recovery (23). EPO induces the cardioprotective effect in two phases of acute and delayed (13), that confirms the implication of multiple signaling pathways for the cardioprotective effect of EPO. Several mechanisms are described for the anti-ischemic effect of EPO. This effect has been attributed to its anti-apoptotic, anti-oxidative and anti-inflammatory properties (10). It is believed that EPO acts directly as scavenger through its sugar-moiety(25). It also activates the antioxidant enzyme such as catalase, superoxide dismutase and glutathione peroxidase. Based on the known role of ROS formation and oxidative stress in cell injury following the CO poisoning (5, 6), we may speculate that the antioxidative effect of EPO could be a mechanism for its observed protective effect on CO cardiotoxicity in this study.

In the present study, for the first time, the effect of EPO was evaluated on the cardiotoxicity of CO poisoning. EPO has significantly attenuated ischemic changes of ECG induced by CO poisoning. It should be noticed that while these results are obtained from EPO administration after CO intoxication, EPO could be evaluated as a treatment agent for CO poisoning. Currently, the molecular and cellular mechanisms of EPO protective effects on CO cardiotoxicity are being studied in our laboratory.

## Conclusion

Due to the importance of cardiotoxicity in CO poisoning and its role in the morbidity and mortality of CO poisoning, an effective treatment is needed for reducing these complications. For the first time, results of our study have indicated the effect of EPO on reducing cardiac ischemia following CO poisoning. More studies are needed to evaluate the effect of EPO on cardiac function parameters due to CO intoxication. Furthermore, it would be valuable to study the EPO effect on ventricular arrhythmia and fibrillations following more severe CO poisoning.
